# Reply to “Validating the psychomotor vigilance task cutoff for residual excessive daytime sleepiness in treated obstructive sleep apnea”

**DOI:** 10.1007/s44470-026-00115-6

**Published:** 2026-07-01

**Authors:** Barbara Junco, Alberto Ramos, Roger McIntosh

**Affiliations:** 1 Department of Neurology, University of Miami, Miller School of Medicine, Miami, FL USA; 2 Department of Psychology, University of Miami, Coral Gables, FL USA; 3 Clinical and Translational Science Institute, University of Miami, Coral Gables, FL USA

We thank Fei and colleagues for their response [1] to our work, “Cognition and psychomotor vigilance in treated sleep apnea patients with and without daytime sleepiness: the MAGNETO study” [2], and for raising questions about the clinical relevance of the PVT cutoff used to define residual EDS. Their letter provides an opportunity to clarify aspects of our methodology that directly address their concerns.

First, the MAGNETO study was designed as an exploratory, hypothesis generating analysis of an underrepresented sample of primarily Hispanic/Latino adults, as described in the published article [2]. The study was not intended as a validation study for the PVT cutoff or presented as such. While the > 5 lapse threshold adopted from prior OSA literature in PAP-adherent populations was not validated against cognitive or clinical measures, its adoption was intended to enable comparison with extant findings on residual EDS and its neurological correlates [3, 4].

Second, we agree that sensitivity analyses using alternative PVT lapse thresholds support the robustness of the > 5 lapse findings. In fact, these analyses are already presented in Supplemental Table S2 of the published article [2]. Those analyses demonstrate that the primary findings remained robust across alternative cutoff values, addressing the concern about threshold specificity.

Third, the primary analytic approach in the MAGNETO study was continuous, not categorical. Total lapses, mean reaction time, and reaction time coefficient of variation (RTCV) were modeled as continuous predictors in regression analyses adjusted for sex, time since diagnosis, and self-reported sleep duration. The categorical EDS grouping was used for descriptive and supplementary purposes only.

Nonetheless, we agree with Fei and colleagues that ROC-based cutoff validation targeting cognitive endpoints and standardized reporting of continuous PVT metrics are worthwhile directions for future EDS research, particularly as larger samples become available. We appreciate their constructive commentary.

## Data Availability

Not applicable.

## References

[1] Fei X, Yang M, Du H, Liu Q. Validating the psychomotor vigilance task cutoff for residual excessive daytime sleepiness in treated obstructive sleep apnea. J Clin Sleep Med. 2026;22(1). 10.1007/s44470-026-00097-5.42260045 10.1007/s44470-026-00097-5PMC13246986

[2] Junco B, Ramos A, Hernandez-Cardenache R, et al. Cognition and psychomotor vigilance in treated sleep apnea patients with and without daytime sleepiness: the MAGNETO study. J Clin Sleep Med. 2026;22(1):60. 10.1007/s44470-026-00077-9.41991883 10.1007/s44470-026-00077-9PMC13087004

[3] Xiong Y, Zhou XJ, Nisi RA, et al. Brain white matter changes in CPAP-treated obstructive sleep apnea patients with residual sleepiness. J Magn Reson Imaging. 2017;45(5):1371–8. 10.1002/jmri.25463.27625326 10.1002/jmri.25463PMC5350066

[4] Zhang J, Weaver TE, Zhong Z, et al. White matter structural differences in OSA patients experiencing residual daytime sleepiness with high CPAP use: A non-Gaussian diffusion MRI study. Sleep Med. 2019;53:51–9. 10.1016/j.sleep.2018.09.011.30445240 10.1016/j.sleep.2018.09.011PMC6360120

